# Sarcomatoid renal cell carcinoma: a case report and literature review

**DOI:** 10.1186/s12882-018-0884-7

**Published:** 2018-04-10

**Authors:** Xiang Liang, Yupin Liu, Pengcheng Ran, Meili Tang, Changlei Xu, Yazhen Zhu

**Affiliations:** 1 Department of medical imaging, Guangdong Provincial Hospital of Chinese Medicine, No.55, Neihuan Rd. W., Guangzhou Higher Education Mega Center, Panyu District, Guangzhou, 510006 Guangdong Province China; 2 Department of pathology, Guangdong Provincial Hospital of Chinese Medicine, No.55, Neihuan Rd. W., Guangzhou Higher Education Mega Center, Panyu District, Guangzhou, 510006 Guangdong Province China

**Keywords:** Sarcomatoid renal cell carcinoma, Imaging features, Targeted therapies

## Abstract

**Background:**

The poorly differentiated renal cell carcinoma (RCC) with rhabdomyosarcomatous sarcomatoid differentiation shows a severely aggressive biological behavior characterized by rapid disease progression. Preoperative identification of the subtype with the prognostic factors and imaging features of sarcomatoid renal cell carcinoma (SRCC) would be of great clinical significance.

**Case presentation:**

A 45-year-old male patient presented a nine day history of gross hematuria without any other symptoms. A computed tomography (CT) and a full-body fluorine-18 fluoro-2-deoxyglucose (FDG) positron emission tomography (PET) - computed tomography (CT) scan urogram were performed. An initial diagnosis identified a space-occupying lesion of the right kidney, retroperitoneal and right renal hulum lymph node metastases, as well as a space-occupying lesion of the third thoracic vertebra (T3). A right radical nephrectomy was performed. Pathologic analysis revealed poorly differentiated RCC with rhabdomyosarcomatous sarcomatoid differentiation that extends into the renal sinus and the ureteral (T3N1M1). Five days later, the Magnetic Resonance imaging (MRI) evidenced a diffused osseous metastatic disease in the thoracic and lumbar vertebra and multiple retroperitoneal lymph node metastases. The disease progressed quickly to multiple organ dysfunction syndrome (MODS) in half a month and the patient died of respiratory failure two days later. The patient refused any chemoradiotherapy in the hospital.

**Conclusions:**

Our case presents a SRCC with severe, aggressive, and rapid disease progression. Classifying SRCC imaging features by CT, MRI as well as PET-CT techniques could potentially be helpful for preoperative identification of the subtype. The prognostic factors of SRCC would be of great clinical interest.

## Background

Sarcomatoid renal cell carcinoma (SRCC) is a form of dedifferentiated renal cell carcinoma (RCC) with aggressive behavior. Preoperative identification of the subtype and prognostic factors of SRCC would be of great clinical significance. The aim of this paper is to present a rare case of a 45-year-old male patient with SRCC. The poorly differentiated renal cell carcinoma (RCC) with rhabdomyosarcomatous sarcomatoid differentiation exhibits aggressive, rapid disease progression. The prognostic factors and imaging features are presented, − as well as a review of the literature.

## Case presentation

**Fig. 1:**
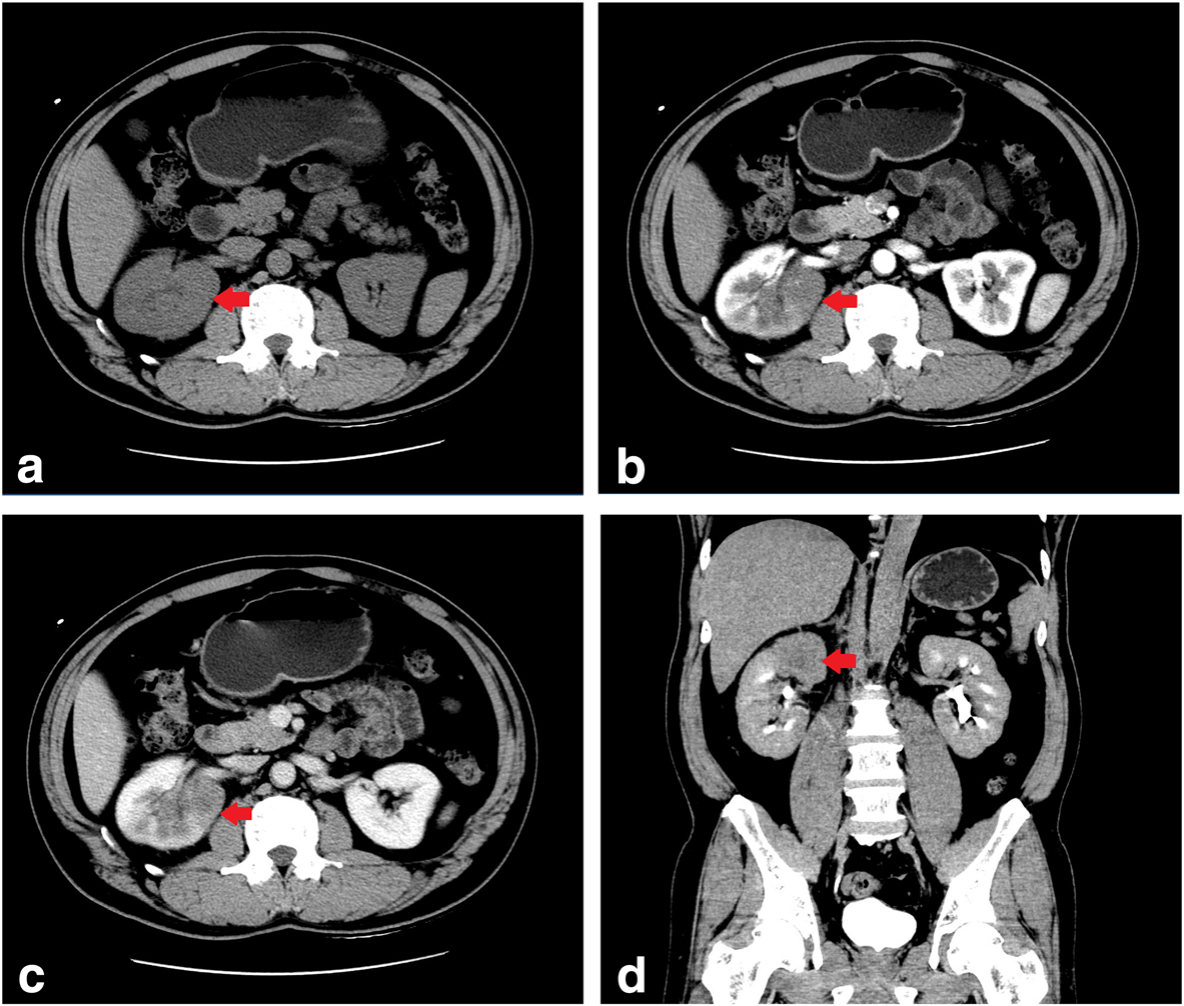
A computed tomography (CT) urogram was performed. Axial and coronal computed tomographic images of the abdomen demonstrating a 6.0 × 4.7 cm irregular mass centered within the superior pole of the right kidney. On the axial noncontrast image (**a**), the mass is non-homogeneous with the most portions being isodense to the normal renal parenchyma and with central hypoattenuating areas suggestive of necrosis. During the corticomedullary phase (**b**), the nephrographic phase (**c**) and the coronal excretory phase (**d**), the solid component of the mass mild-moderately enhances but not as avidly as normal renal cortex. The central low density necrotic areas and the margin become more conspicuous

**Fig. 2:**
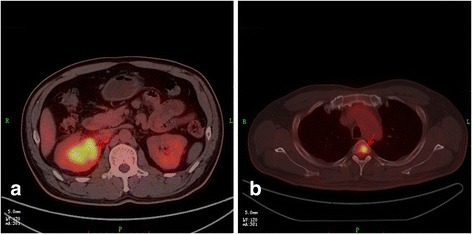
A positron emission tomography (PET) - computed tomography (CT) scan was made. Evidence of intense FDG uptake in irregular mass centered within the superior pole of the right kidney (**a**, arrow) well as diffuse osseous metastatic disease in third thoracic vertebra (**b**, arrow)

**Fig. 3:**
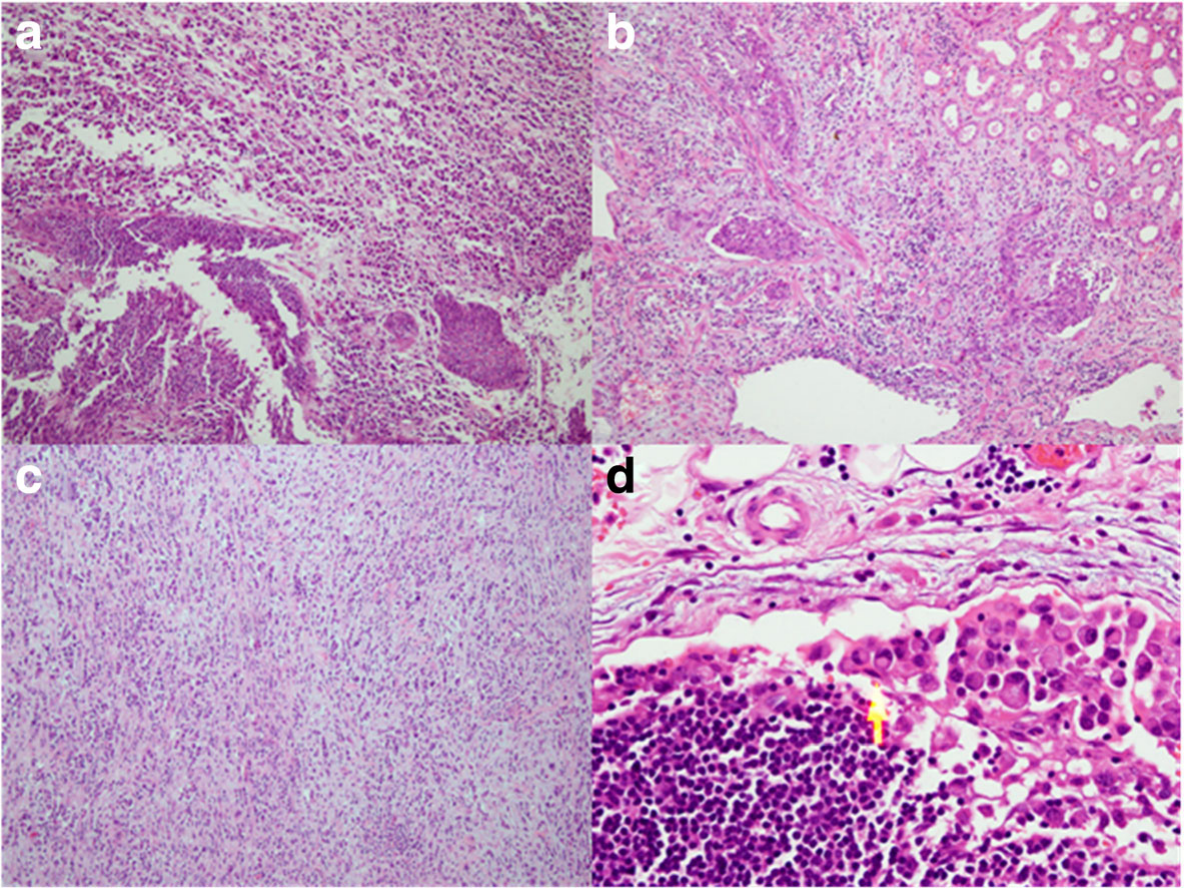
Pathological results. **a**-**c** Section shows infiltrative growth renal cell carcinoma cells with the poorly differentiated (H&E 100X), **d** Section show RCC cells with rhabdomyosarcomatous sarcomatoid differentiation (arrow, H&E 400X)

**Fig. 4:**
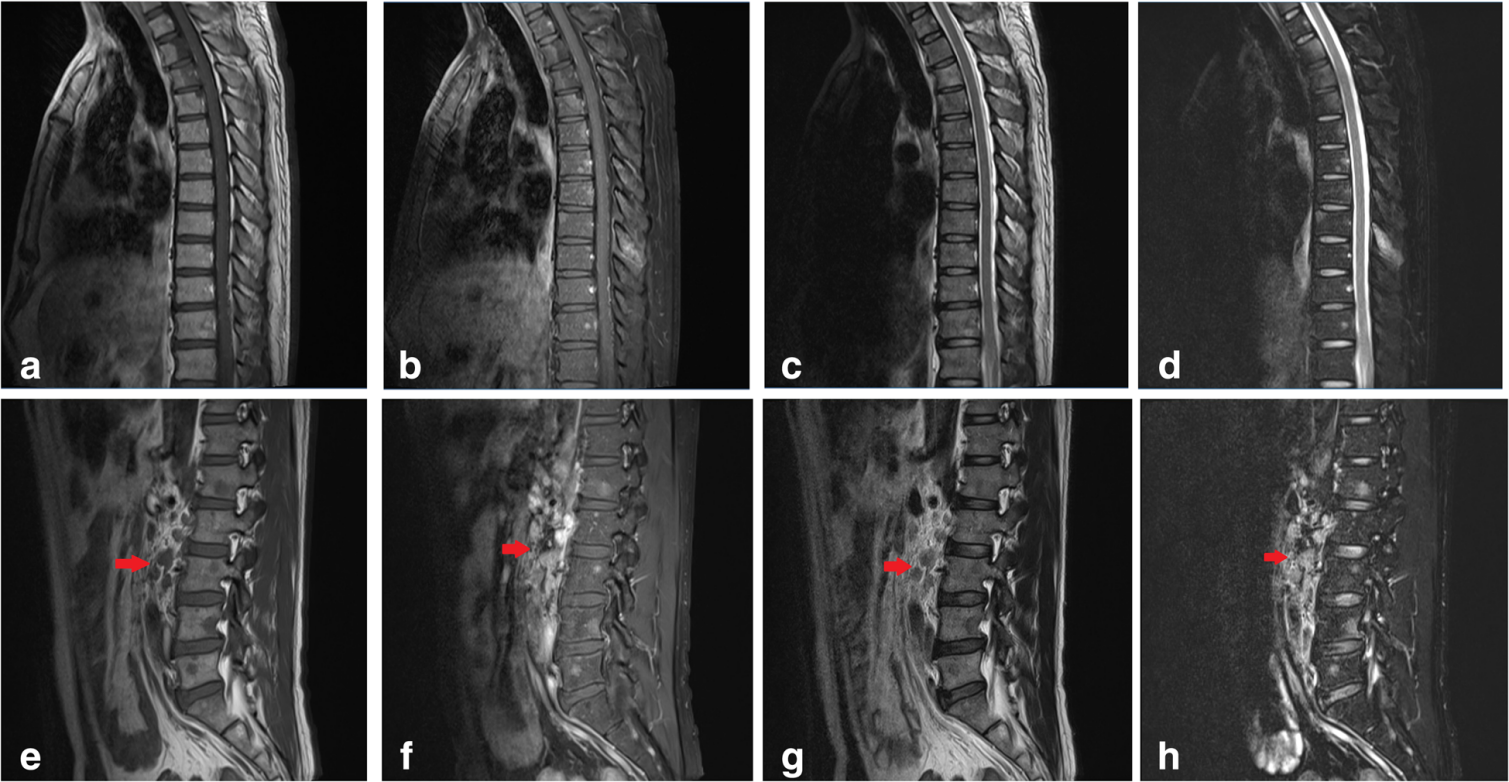
The magnetic resonance imaging (MRI) of the thoracic and lumbar vertebra was performed. Evidence of diffuse osseous metastatic disease in the thoracic and lumbar vertebra and multiply retroperitoneal lymph node metastases which were mixed together as masses (arrow). **a**-**d**: imaging of thoracic spine; **a**: T1 weighted imaging (T1WI); B: T1WI + FS (fat suppressed sequence); **c**: T2 weighted imaging (T2WI); **d**: T2WI + FS. **e**-**h**: imaging of lumbar spine; **e**: T1WI; **f**: T1WI + FS; **g**: T2WI; **h**: T2WI + FS

A 45-year-old male patient presented a nine-day history of gross hematuria without any symptoms. Physical and laboratory examination revealed no significant abnormalities except for microscopic hematuria. The results of a computed tomography (CT) urogram demonstrated a large non-homogeneous mass of 6.0 × 4.7 cm within the superior pole of the right kidney, (with extension into the renal sinus). The mass is heterogeneous with the peripheral portions being isodense to the normal renal parenchyma and with central hypoattenuating areas suggestive of necrosis. The lesion exhibited mild to moderate enhancement on all three phases of post-contrast imaging (corticomedullary, nephrographic and excretory phases) (Fig. 1). There are several prominent retroperitoneal (left para-aortic) lymph nodes, measuring up to 0.9 cm in short axis diameter. In addition, recommendations for a full body fluorine-18 fluoro-2-deoxyglucose (FDG) positron emission tomography (PET) - computed tomography (CT) scan were made by urology and oncology to assist with diagnosis and the staging of the malignancy. The PET-CT confirmed a large renal mass with intense FDG uptake (SUVmax was up to 8.8) and a single osseous metastatic disease in third thoracic vertebra (T3) (Fig. 2). Several small lymph nodes with slight FDG uptake were also found in prominent retroperitoneal (left para-aortic), and right renal hulum. An initial diagnosis of a space-occupying lesion of the right kidney, retroperitoneal and right renal hulum lymph node metastases and a space-occupying lesion of the T3 was proposed with suspicions of renal cell carcinoma and subtypes or lymphoma. Subsequently, the patient underwent right radical nephrectomy. One mass was revealed in the superior pole of the kidney, which extends locally into the capsule and micrometastasis was found in 10 lymph node of right renal hulum. The renal vein and adrenal gland is normal and free of tumor. Despite the lack of histologic evidence within the biopsied material to determine a specific subtype of RCC, pathologic analysis revealed poorly differentiated renal cell carcinoma (RCC) with rhabdomyosarcomatous sarcomatoid differentiation, extending into the renal sinus and the ureteral (T3N1M1) (Fig. 3). Subsequently, the patient refused any chemoradiotherapy and left the hospital without any obvious symptoms. However, five days later, due to lumbago and weakness, the patient returned to the hospital. The Magnetic Resonance imaging (MRI) indicated diffuse osseous metastatic disease in the thoracic and lumbar vertebra and multiple retroperitoneal lymph node metastases, which mixed together as masses (Fig. 4). The patient refused any chemoradiotherapy again. The disease progressed quickly to multiple organ dysfunction syndrome (MODS) in half a month. Eventually, the patient died of respiratory failure two days later.

## Discussion

RCC is the most common malignant epithelial tumor of the kidney in adults, with the three major subtypes of clear cell RCC, papillary RCC, and chromophobic RCC, accounting for 75%, 15%, and 5% of all renal neoplasms, respectively [1]. Initially considered as a renal sarcoma, sarcomatoid renal cell carcinoma (SRCC) is now recognized as a form of dedifferentiated carcinoma and is therefore not a distinct histologic entity. SRCC presents RCC in which a portion of the tumor undergoes transformation into a high-grade undifferentiated component that is characterized by the presence of spindle cells, but the tumors characteristically show both epithelial and mesenchymal differentiation by ultrastructural and immunohistochemical analyses [2, 3].

In addition, the epithelial and sarcomatoid components of SRCC largely contain the same genomic features, suggesting a common cell of origin [4, 5]. The average incidence of SRCC is 8% among all RCCs [2]. It is well known that patient with SRCC is associated with poor prognosis, regardless of the underlying RCC subtype.

Other publications have reported a median survival time of only 4-9 months after diagnosis [6]. Only a few patients with early-stage disease (stage I and stage II) demonstrated extended survival [2]. In this case, the SRCC shows a greatly aggressive biological behavior characterized by rapid disease progression. When the patient returned to the hospital, the MRI indicated diffuse osseous metastatic disease in the thoracic and lumbar vertebra and multiple retroperitoneal lymph node metastases, which were mixed together as masses.

The findings indicated the progression of disease compared with a single osseous metastatic disease in T3 and several small lymph nodes in prominent retroperitoneal (left para-aortic), and right renal hulum in PET-CT imaging. It was just half a month between the MR imaging and PET-CT imaging. Simultaneously, the disease progressed quickly to the MODS within half a month. Finally, the patient died of respiratory failure two days later. The patient survived approximately for one month after the diagnosis and disease onset.

Major prognostic factors of SRCC should emphasize the proportionality of sarcomatoid components, pathological stage, tumor necrosis, tumor size and genetic factors. A predictor and possibly catalyst of multiple acute diseases, sacomatoid components have inspired, several studies to prove that increased SRCC worsens the chance of survival [2]. In addition, multiple studies confirmed the tumors with high TNM (Tumor Node Metastasis) stage to be a predictor of poor survival [2]. In high TNM stage, distant metastases most frequently migrate to the lungs and bones and are associated with poor prognosis [7].

Tumor necrosis represents a further important prognostic feature. Tumors with increased necrosis in the sarcomatoid areas have lowered survival rates, while the amount of histologic tumor necrosis is not associated with outcome [7]. Tumor size may also be partly responsible for prognosis, which was identified as independent predictor of cancer-specific mortality [8].

Finally, ATrich interaction domain 1A (ARID1A) and BRCA1 associated protein 1 (BAP1) somatic single-nucleotide variants (SSNVs) were exclusive to sarcomatoid components and deficiency of ARID1A and BAP1 has been associated with worse prognosis, higher tumor grade, and a higher incidence of sarcomatoid histology [5]. Our case presents a disease of advanced stage. The disease extended into the renal capsule and right renal hulum. The lymph node metastases and T3 metastases manifested themselves. The necrosis in the tumor was observed. Therefore, the sarcomatoid component and tumor necrosis may partly attest to intensely aggressive biological behavior characterized by rapid disease progression in this case.

The SUVmax potently serves as a novel biomarker to predict the survival time of patients with advanced RCC. Recently, several studies have found high SUVmax values are associated with bigger tumor size, higher GLUT expression, higher nuclear grade and less necrosis in patients with RCC [9]. In this case, the SRCC with high SUVmax shows increased aggressive biological behavior. The value of SUVmax for SRCC and its prognosis require larger groups of patients to be examined.

The patients with preoperatively identified SRCC were recommended to reserve surgery and undergo upfront systemic therapy [3]. And the patients with inoperable or metastatic RCC typically should undergo systemic treatment with targeted agents and/or immune checkpoint inhibitors [1]. Recently, several studies described the outcome and prognosis of metastatic SRCC in the targeted therapy era. Voss et al. reported that patients with metastatic sarcomatoid CRCC can benefit from mTOR-targeted therapy using rapalog-type mTOR inhibitors, but the majority of patients respond poorly with these agent, with > 50% suffering progressive disease as their best response to treatment [10].

Another retrospective study indicates that, despite the tremendous progress in the treatment of metastatic RCC with VEGF inhibitors, the prognosis of SRCC remains poor with greater risk of relapse, worse baseline prognostic criteria, and worse clinical outcome [11]. Thus, additional insight into the biology of SRCC is needed to develop alternative therapeutics. Current research into genomic characterization of SRCC suggests that therapeutic strategies may include cell cycle inhibitory agents or synthetic lethal strategies to overcome the loss of TP53, CDKN2, and NF2, as revealed by genomic profiling catastrophic genomic events in a tumor [4]. The mTOR-targeted therapy may improve the prognosis of SRCC in NF2-deficient tumors [4]. In addition,TP53 and ARID1A represent potential sarcomatoid-specific targets for which therapeutics are in development [5]. An age of onset of ≤46 years raises the possibility of a hereditary syndrome in RCC [1]. With a mean age of patients with SRCC at approximately 60 years, genetic defects represent causes of sarcomatoid differentiation in this 45-year-old patient.

As mentioned above, preoperative identification of SRCC would help determine the therapeutic strategies [3]. However, as current biopsy and radiographic identification may not accurately identify patients with a limited sarcomatoid component and the lack of specific clinical features make preoperative identification challenging, thus different strategic approaches must be developed [3].

Several researchers have reported the usefulness of CT or MRI for differentiating SRCC from other RCC subtypes. Nicola Schieda et al. reported the features of CT findings, including texture analysis, which can differentiate SRCC from CRCC [12]. The study showed larger tumor size of SRCC than CRCC overall, an increased frequency of peritumoral neovascularity and an increased size of peritumoral neovascularity in SRCC when compared with CRCC [12]. In texture analysis, the global heterogeneity features (lengthwise nonuniformity and greater gray-level nonuniformity) manifested in SRCC more than in CRCC [12]. Takeuchi et al. evaluated findings of SRCC on MRI and found that the presence of low signal intensity (SI) on T2-weighted images (T2LIA) corresponding to the area showing a hypovascular nature and markedly restricted diffusion might be characteristic findings of SRCC [13]. In the study, nodules with low SI within a heterogeneous mass with high SI and a mixed or separated pattern of high SI on T2-weighted images (T2HIA) and T2LIA were characteristic findings of SRCC dedifferentiated from CCRC [13]. It is pathologically confirmed that T2HIA and T2LIA on MRI roughly corresponded to CCRC and SRCC, respectively [13]. In another study, Takeuchi et al. defined heterogeneous renal tumors with 1–3 cm T2LIAs as a probable SRCC finding and tumors with > 3 cm T2LIAs, > 1 cm multiple T2LIAs, or disruption of the pseudocapsule as definite SRCC findings to investigate the usefulness of MRI for detection of SRCC components within RCC and differentiation from other renal tumors [14]. Intratumoral hemorrhage and necrosis were seen, but these imaging features are non-specific [13, 15]. Recently, for an integrated diagnostic approach between radiology and genomics, the term radiogenomics has been established [16]. Karlo et al. preliminary revealed associations of clear cell RCC between CT features and underlying mutations by radiogenomics analysis [16]. The findings are necessary to warrant further investigation and validation.

## Conclusions

In conclusion, the present study reported one case of SRCC with a high degree of malignancy and rapid progress. The imaging features of SRCC include an ill-defined, non-envelope soft-tissue mass with slight mass effect, not clearly demarcated from renal parenchyma and not enhanced as avidly as periphery renal parenchyma. Additionally, in this case, PET-CT is effective for detecting malignancy with high SUVmax and restaging in SRCC. Therefore, it seems that the utility of CT and MRI for differentiating SRCC from other RCC subtypes and PET-CT will play an important role in detecting malignancy, primary staging/restaging, therapeutic decision-making, detecting recurrence, assessing residual masses and monitoring therapeutic response in SRCC. Considering that histologic subtype of RCC has been strongly correlated with prognosis, the clinical implications and therapeutic strategies, as well as the preoperative identification of the subtype and the prognostic factors of SRCC through imaging would be of great clinical interest. Further studies and a larger case series will be required to fully clarify SRCC imaging features by CT, MRI as well as PET-CT techniques. A possible approach is to combine individual imaging features into imaging phenotypes of RCC and correlate them to underlying genomic profiles.
